# Preclinical evaluation of a next-generation, subcutaneously administered, coagulation factor IX variant, dalcinonacog alfa

**DOI:** 10.1371/journal.pone.0240896

**Published:** 2020-10-28

**Authors:** Timothy C. Nichols, Howard Levy, Elizabeth P. Merricks, Robin A. Raymer, Martin L. Lee

**Affiliations:** 1 Department of Pathology and Laboratory Medicine, University of North Carolina at Chapel Hill, Chapel Hill, North Carolina, United States of America; 2 Catalyst Biosciences, South San Francisco, California, United States of America; 3 Department of Biostatistics, UCLA Fielding School of Public Health, Los Angeles, California, United States of America; University of Pennsylvania Perelman School of Medicine, UNITED STATES

## Abstract

**Introduction:**

The rapid clearance of factor IX necessitates frequent intravenous administrations to achieve effective prophylaxis for patients with hemophilia B. Subcutaneous administration has historically been limited by low bioavailability and potency. Dalcinonacog alfa was developed using a rational design approach to be a subcutaneously administered, next-generation coagulation prophylactic factor IX therapy.

**Aim:**

This study aimed to investigate the pharmacokinetic, pharmacodynamic, and safety profile of dalcinonacog alfa administered subcutaneously in hemophilia B dogs.

**Methods:**

Two hemophilia B dogs received single-dose daily subcutaneous dalcinonacog alfa injections for six days. Factor IX antigen and activity, whole blood clotting time, and activated partial thromboplastin time were measured at various time points. Additionally, safety assessments for clinical adverse events and evaluations of laboratory test results were conducted.

**Results:**

There was an increase in plasma factor IX antigen with daily subcutaneous dalcinonacog alfa. Bioavailability of subcutaneous dalcinonacog alfa was 10.3% in hemophilia B dogs. Daily subcutaneous dosing of dalcinonacog alfa demonstrated the effects of bioavailability, time to maximal concentration, and half-life by reaching a steady-state activity sufficient to correct severe hemophilia to normal, after four days.

**Conclusion:**

The increased potency of dalcinonacog alfa facilitated the initiation and completion of the Phase 1/2 subcutaneous dosing study in individuals with hemophilia B.

## Introduction

Hemophilia B is a hereditary X-linked bleeding disorder caused by factor IX (FIX) deficiency [1]. Hemophilia B, characterized by frequent (in severe phenotypes) and spontaneous bleeding [1, 2] into joints, muscles, and body cavities, can lead to arthropathy with progressive cartilage damage, chronic pain, disability, diminished quality of life and ultimately joint destruction [1]. Disease classification of mild, moderate, or severe phenotype is based on residual plasma FIX levels [3, 4]. Approximately 60–70% of patients have a moderate or severe form [5–7]. Historically, treatment has been on demand, (using clotting FIX replacement therapy administered when a hemorrhage occurs), or before surgery [8]. Currently, the standard of care treatment is intravenous FIX replacement therapy administered prophylactically, at regular intervals to maintain adequate FIX levels and prevent the onset of bleeding episodes [8–11]. Routine prophylaxis can substantially reduce bleeding episodes [11–13], prevent joint diseases [11, 14] and reduce the risk of death, but unfortunately, many patients do not receive prophylaxis and need to treat bleeding episodes on-demand [15, 16]. Treatment with FIX products, namely recombinant human FIX (rFIX), requires two or three intravenous (IV) infusions per week to achieve effective bleeding prevention due mostly to the short half-life of FIX [17–21]. A high frequency of prophylactic infusions can be a major adherence barrier, especially in pediatric patients, and those with poor venous access [22–24]. Extended half-life FIX products have recently become available. These require approximately weekly IV infusions, and some have reduced extravascular distribution that has been associated with reduced prevention of bleeding [25, 26]. A subcutaneous FIX product with a longer half-life to prolong the protective hemostatic effect would reduce the need for repeated venous access. This could potentially improve the acceptance of prophylactic regimens and adherence by patients with hemophilia. Subcutaneous administration could be the preferred route of administration for convenience and less pain but has been limited historically by low bioavailability and potency [27–31].

**Fig 1:**
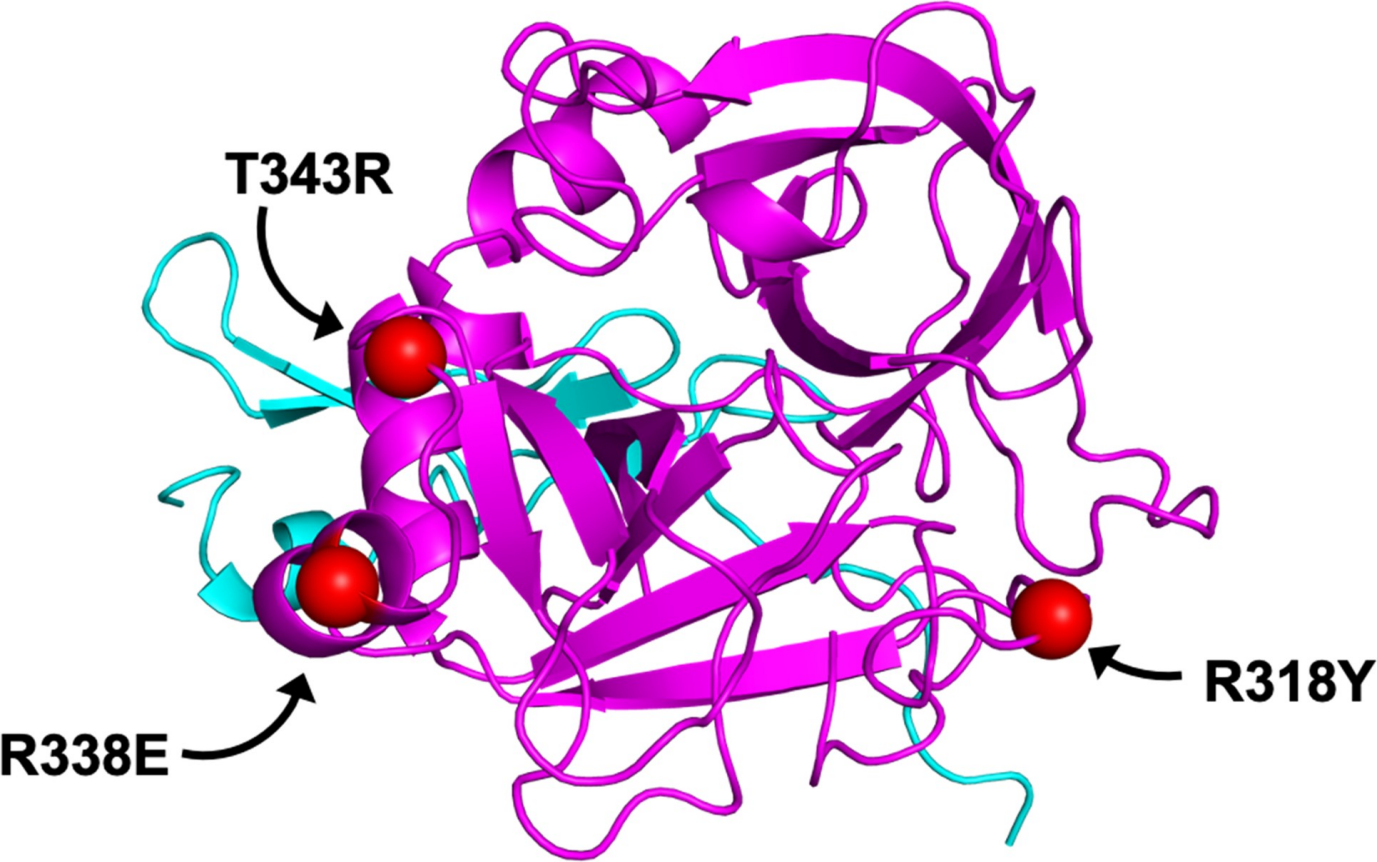
Dalcinonacog alfa has three substitutions: R318Y/R338E/T343R. Structural representation of the three substitutions in dalcinonacog alfa modeled onto PDB 1RFN [38]. The three amino acid substitutions are shown as red spheres and found in two conserved loops within the serine protease domain for FIX, namely R318Y located in the ‘150-loop’ provides resistance to inhibition by antithrombin, R338E and T343R, both located in the ‘170-loop’, provide enhanced procoagulant activity and affinity for activated FVIII.

Dalcinonacog alfa (also known as CB 2679d), was designed using a rational design approach to be a next-generation of coagulation FIX administered subcutaneously for prophylactic therapy. Dalcinonacog alfa has three amino acid substitutions in two loops within the FIX protein (**Fig 1**): By way of the mature FIX sequence numbering, (1) R318Y located in the ‘150-loop’, stabilizes activated FIX (FIXa), directly interacts with the substrate factor X (FX) and provides resistance to antithrombin; (2) R338E, and, (3) T343R, both located in the ‘170-loop’, significantly enhance affinity to the cofactor, activated factor VIII (FVIIIa) and increase the catalytic activity FIXa [32]. For clarity and reference to other nomenclature, R318Y/R338E/T343R refer to R150Y/R170E/T175R in classic chymotrypsin numbering [33] and R364Y/R384E/T389R in the Human Genome Variation Society (HGVS) nomenclature, which includes the 46 amino acid propeptide [34]. These molecular structure substitutions enable dalcinonacog alfa to increase catalytic activity (dalcinonacog alfa has demonstrated 3-times the catalytic efficiency to FX [substrate]); increase affinity for FVIIIa (dalcinonacog alfa has a 10-times higher affinity to cofactor FVIIIa) and improve resistance to inhibition by antithrombin (dalcinonacog alfa has 15-times the resistivity to inhibitor antithrombin compared to wild-type FIX [wt-FIX]), with a resultant 20-fold enhanced potency *in vitro* (clotting activity) and *in vivo* (tail clip model) and 8-fold increased duration of aPTT activity *in vivo* compared with recombinant wt-FIX dosed at the same mass [35, 36]. Additional preclinical studies found that the pharmacokinetic profile of dalcinonacog alfa was similar to BeneFIX when dosed using the same mass; however, dalcinonacog alfa has approximately 22-times greater potency and, therefore, can achieve higher activity at an equal mass dosing level [37].

The aim of this preclinical study was to evaluate the pharmacokinetics (PK), pharmacodynamics (PD), and safety of dalcinonacog alfa in hemophilia B dogs after daily subcutaneous administration.

## Materials and methods

### Study drugs

Dalcinonacog alfa is produced by a recombinant Chinese hamster ovary cell clone in suspension culture that coexpresses rFIX and recombinant human wild-type furin. Dalcinonacog alfa was injected at single subcutaneous doses of 42, 72, 125, and 143 IU/kg, followed by intravenous 50 IU/kg. Subsequently, 300 IU/kg was injected subcutaneously daily for six days. Dalcinonacog alfa was engineered by Catalyst Biosciences, Inc. and ISU Abxis manufactured the drug for this study (Lot#E1601 Y, Sample ID: ISU304 6/9/16). ISU Abxis and Catalyst Biosciences, Inc. collaborated on the pre-clinical and early clinical development for dalcinonacog alfa.

### Animals

Scarce hemophilia B dogs that had not previously been exposed to human clotting factors were chosen to characterize the PK, PD, and safety profile of dalcinonacog alfa. Canine models of hemophilia B are extensively used and are highly predictive to evaluate the PK and efficacy of human coagulation factors, due to the similarities with regard to severe bleeding phenotype and hemostatic protein PK [39–41]. Specifically, the PK, PD, and clinical efficacy of recombinant clotting factor concentrates were accurately predicted by pre‐clinical dog studies [42]. Animals were bred and maintained at the Francis Owen Blood Research Laboratory (FOBRL) at the University of North Carolina (UNC) at Chapel Hill, NC. Dogs at the FOBRL are housed in dedicated indoor-outdoor runs that meet or exceed USDA space requirements. The FOBRL is inspected by the UNC Institutional Animal Care and Use Committee (IACUC) semi-annually, the United States Department of Agriculture (USDA) annually and Association for Assessment and Accreditation of Laboratory Animal Care International (AAALACi) every three years. The FOBRL has consistently passed all of these inspections including those that occurred during the course of this study. Food is provided once or twice a day to maintain an acceptable body condition and appropriate weight as determined by the attending veterinarians. Water is provided ad lib. Environmental enrichment is provided by the Division of Comparative Medicine personnel who specialize in this field and who work with the FOBRL husbandry staff to deliver enrichment. The enrichment program includes, but is not limited to, daily interaction with other dogs and with humans, outdoor exercise in dedicated play yards, paired housing, a wide variety of chew toys and treats such as milk bones. This study was conducted in four hemophilia dogs (Tony, Bennet, P07, and P41 –**Table 1**) and was carried out in strict accordance with the recommendations in the Guide for the Care and Use of Laboratory Animals of the National Institutes of Health. The mutation in the Chapel Hill hemophilia B dogs is a missense mutation (G to A at nucleotide 1477) that results in the substitution of glutamic acid for glycine-379 in the catalytic domain of the FIX molecule [43]. All animal study procedures were approved by the UNC Institutional Animal Care and Use Committee. The protocol was approved by the Committee on the Ethics of Animal Experiments of the UNC. No surgery was performed and all efforts were made to minimize suffering. During the course of this study, the dogs were monitored for activity level several times every day, food and water consumption daily, body weight, daily temperatures, change in hematocrit or complete blood counts and serum chemistry values. The attending veterinarians performed physical exams on these dogs prior to their entry into this study and helped monitor the subcutaneous injection sites during and after the study. No euthanasia was required for this study.

**Table 1:** Hemophilia dogs used in this study.

Dog^a^	Sex	DOB	First Dose	Age at First Dose	Weight at First Dose
Tony	Male	18-Dec-11	11-May-16	4 years and 5 months	23.0 kg
Bennett	Male	24-Apr-14	11-May-16	2 years and 1 month	19.8 kg
P07	Female	10-Mar-11	19-Sep-16	5 years and 6 months	24.5 kg
P41	Male	18-Dec-11	19-Sep-16	4 years and 9 months	21.8 kg

DOB, date of birth; kg, kilogram.

^a^The mutation in the Chapel Hill hemophilia B dogs is a missense mutation (G to A at nucleotide 1477) that results in the substitution of glutamic acid for glycine-379 in the catalytic domain of the Factor IX molecule [43].

### Administration and blood sampling

**Fig 2:**
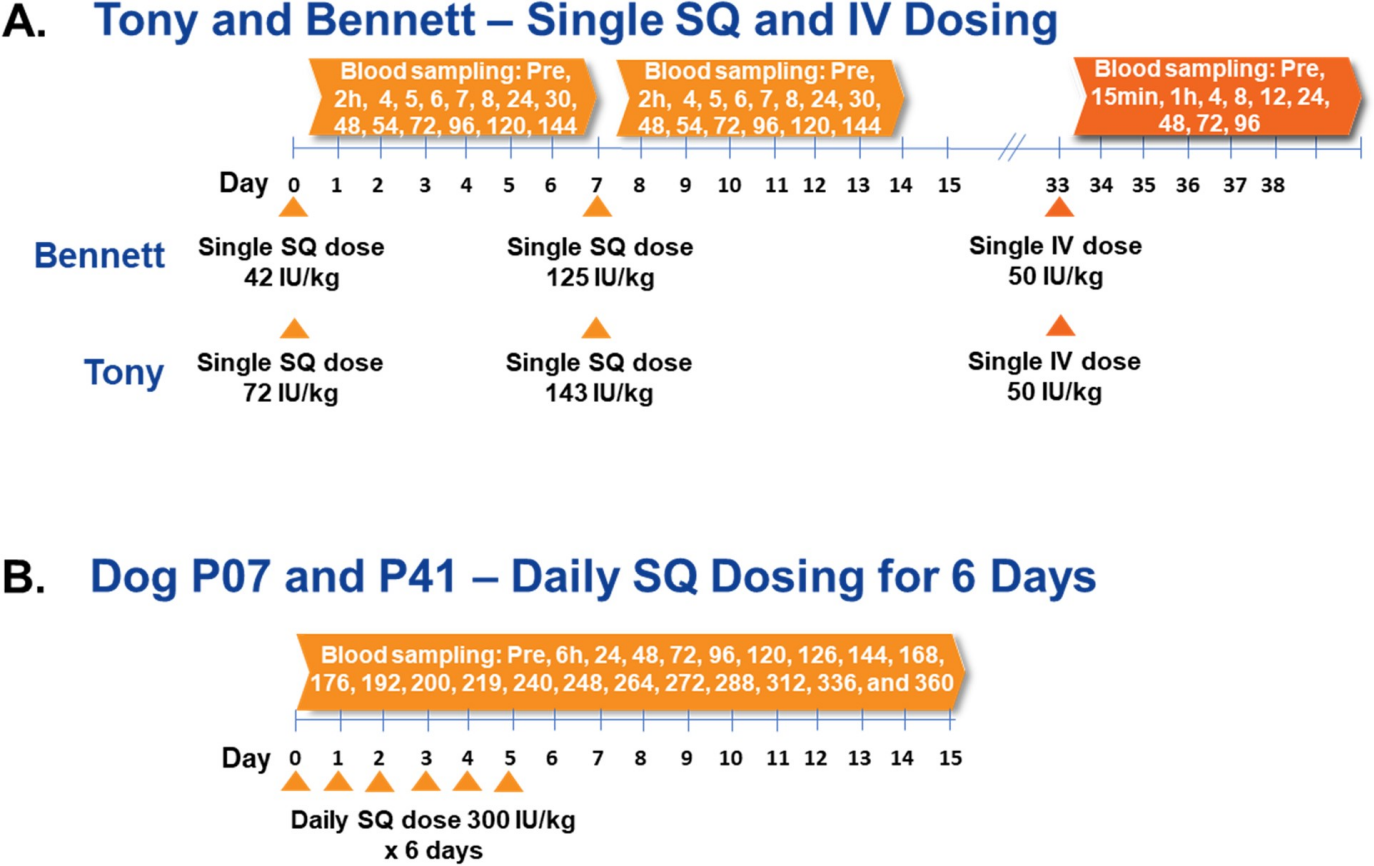
Dalcinonacog alfa administration and blood sampling in 4 dogs. (A) Bennett and Tony received single doses of dalcinonacog alfa injected subcutaneously on Days 0 and 7 and blood was sampled at 2, 4, 5, 6, 7, 8, 24, 30, 48, 54, 72, 96, 120, and 144 hours. Each dog then received 50 IU/kg intravenously and blood was sampled at 0.25, 1, 4, 8, 12, 24, 48, 72, and 96 hours. (B) P07 and P41 received 300 IU/kg of dalcinonacog alfa injected subcutaneously on Days 0, 1, 2, 3, 4, 5 and blood was sampled at 0, 6, 24, 30, 48, 54, 72, 78, 96, 102, 120, 126, 144, 168, 176, 192, 200, 219, 240, 248, 264, 272, 288, 312, 336, and 360 hours. h, hour; kg, kilogram; IV, intravenous; pre, pre-dose; SQ, subcutaneous.

Single doses of dalcinonacog alfa 43 and 125 or 72 and 143 IU/Kg were injected subcutaneously into two dogs (Bennett and Tony) and blood was sampled at 2, 4, 5, 6, 7, 8, 24, 30, 48, 54, 72, 96, 120, and 144 hours. Each dog then received 50 IU/kg intravenously and blood was sampled at 0.25, 1, 4, 8, 12, 24, 48, 72, and 96 hours. 300 IU/kg was administered via subcutaneous daily injection for six days into two other hemophilia dogs (P07 and P41) and blood was sampled at 0, 6, 24, 30, 48, 54, 72, 78, 96, 102, 120, 126, 144, 168, 176, 192, 200, 219, 240, 248, 264, 272, 288, 312, 336, and 360 hours (**Fig 2**). Blood was collected by venipuncture of the cephalic vein (16 to 22 mL of blood collected per time point). The average weight of P07 and P41 was 23.5 kg. We estimate ~80 mL of blood per kg or a total blood volume of ~1,852 mL. Thus, 16 to 22 mL would be ~0.8 to 1.2% of the total blood volume per sample. During the 15-day period, these otherwise healthy dogs are actively replacing with newly synthesized red blood cells. Complete blood counts were performed frequently to monitor for significant drops in hemoglobin or hematocrits, which did not occur.

Venipuncture is performed by experienced personnel who pay careful attention to the need to plan for repeated sampling over the 15-day period. This includes alternating limbs, holding all venipuncture sites until hemostasis is clearly evident and utilizing sites that are at least 1 to 2 cm from any visible previous puncture. In general, hemophilia B dogs weighing at least 20 kg tolerate this number of venipunctures well.

### Determination of FIX antigen and activity

The concentration of rFIX antigen in canine plasma samples was determined using an ELISA (Asserachrom IX:Ag, Diagnostica Stago, cat no 00943). Baseline antigen levels were undetectable [44]. FIX activity was assessed at Haematologic Technologies using an activated partial thromboplastin time (aPTT) Factor IX one-stage clotting assay on an ACL-TOP instrument (Instrumentation Laboratories) using the recommended HemosIL^®^ or SynthasIL^®^ reagents and calibrators.

### Determination of WBCT

Whole blood clotting time (WBCT) was measured at each time point. WBCT was performed using a two‐tube procedure at 28°C [41]. One mL of whole blood collected was distributed equally between two siliconized tubes. The first tube was tilted every 30 seconds. After a clot formed in the first tube, the second tube was tilted and observed every 30 seconds. The WBCT is the clotting time (in minutes) in the second tube.

### Determination of aPTT

Activated partial thromboplastin time (aPTT) was measured at each time point on a STart Hemostasis Analyzer (Diagnostica Stago, Parsippany, NJ) using TriniCLOT Automated APTT reagent and 180-second incubation. The aPTT assay was performed twice, once using a 60‐second incubation and once using a 180‐second incubation.

### Safety assessments

Safety assessments included observation for clinical adverse events and evaluations of laboratory test results and blood samples were collected as outlined previously. This included hematology, chemistry, prothrombin fragment 1+2 (F1+2), d‐dimer, and fibrinogen. No formal inferential statistical analyses were performed.

## Results

**Fig 3:**
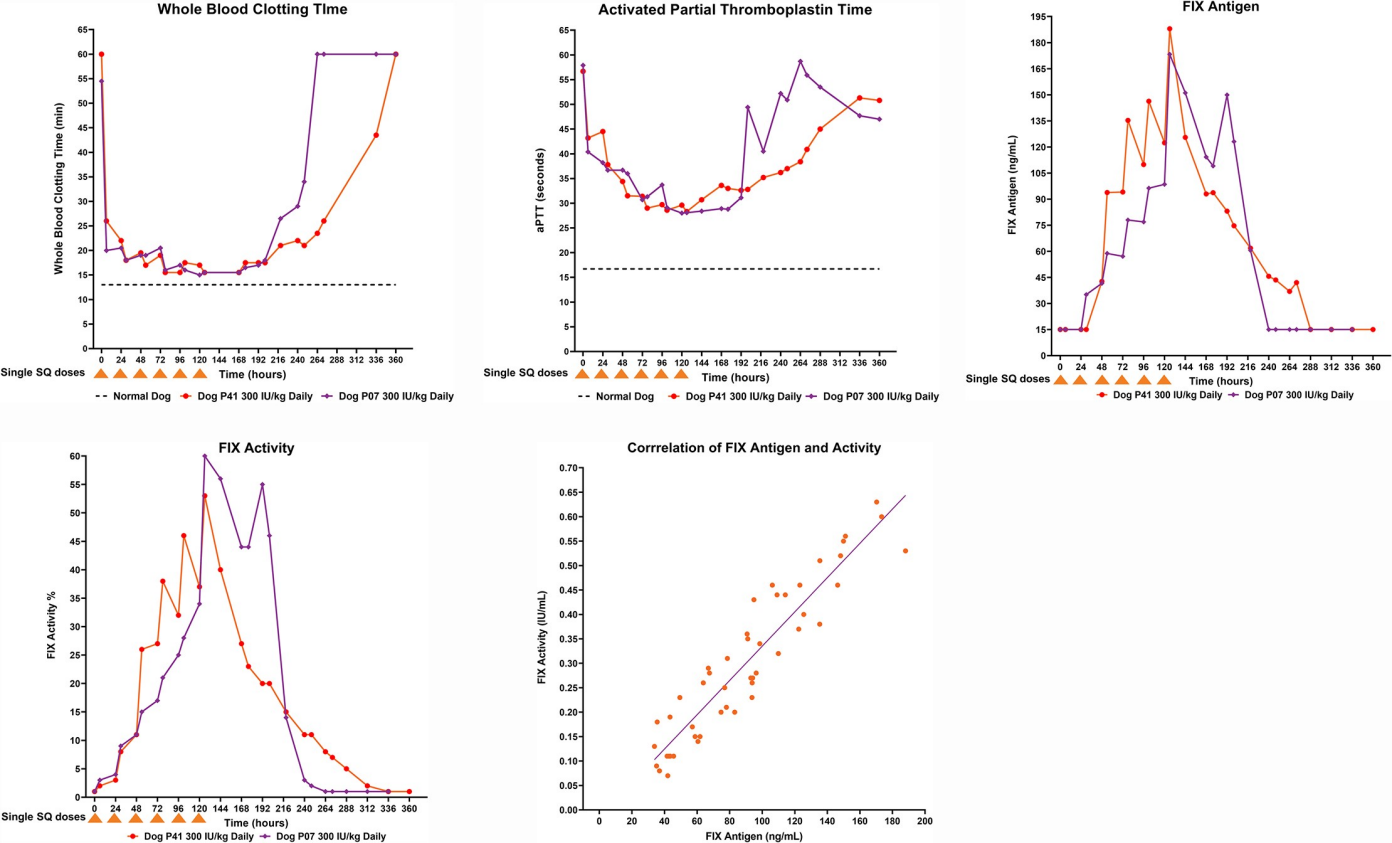
Daily subcutaneous dalcinonacog alfa dosing in hemophilia dogs and consequent blood levels, FIX activity and coagulation results. (A) WBCT; (B) aPTT; (C) FIX antigen; (D) FIX activity; (E) Correlation between FIX activity and antigen. The decrease in aPTT mirrored the reduction in WBCT. There was a progressive increase in plasma FIX antigen with daily subcutaneous injection of dalcinonacog alfa. The plasma FIX activity correlated well with FIX antigen. aPTT, activated partial thromboplastin time; FIX, factor IX; kg, kilogram; Min, minutes; WBCT, whole blood clotting time.

Single subcutaneous doses did not result in any consistent change in WBCT or aPTT and antigen levels were below the level of quantification at all time points. Intravenous dosing confirmed the expected pharmacokinetics for a FIX compound and corresponding shortening of WBCT and aPTT (**S1 Table**). WBCT for P07 and P41 was 54.5 and 60 minutes at baseline (normal <13 minutes) and following daily subcutaneous administration of dalcinonacog alfa decreased to 20 and 26 minutes, respectively, at six hours (**Fig 3A**). The progressive decrease in WBCT reached a nadir of 15 and 15.5 minutes at 120 and 78 hours, respectively. WBCT remained <18 minutes through 200 hours, 80 hours after the last subcutaneous injection. Daily subcutaneous administration of dalcinonacog alfa shortened aPTT in P07 from 57.9 seconds at baseline to 28.0 seconds at 120 hours and in P41 from 46.7 seconds at baseline to 28.3 seconds at 126 hours (**Fig 3B**). For P07 and P41, daily subcutaneous dalcinonacog alfa produced daily fluctuations (peaks and troughs) of dalcinonacog alfa antigen with a progressive increase in both until dosing was completed. The decrease in aPTT mirrored the reduction in WBCT. We do not have sufficient information to explain why the aPTT decreased from 58.7 seconds to 47 seconds after time point 264 hours. Since the FIX:Ag was undetectable at those times, it is likely that this fluctuation in aPTT result is due to background variation in the absence of detectible FIX [45].

There was a progressive increase in plasma FIX antigen with daily subcutaneous injection of dalcinonacog alfa. After six doses there was a peak FIX antigen of 173.3 and 188 ng/mL in dog P07 and P41, respectively (**Fig 3C**). FIX activity is presented in **Fig 3D**. The plasma FIX activity correlated well with FIX antigen; (**Fig 3E**). Bioavailability was determined as the ratio of the dose adjusted area under the curve (AUC) for the SQ infusion relative to that for the IV infusion. The bioavailability of subcutaneous dalcinonacog alfa was 10.3%. The half-life was calculated based on the use of a piecewise log-linear regression that was fit using a robust fitting algorithm (M-regression) [46, 47]. The subcutaneous half-life after daily dosing was 155 hours compared with an IV alpha phase half-life of 28 hours and a beta phase value of 59 hours. Only two dogs were evaluated in this study, which precludes reasonable estimates of the standard errors of the statistical estimates provided.

D-dimer levels remained within the normal range at all times (range in clinically healthy dogs is 0.1 to 0.5 mg/l [48]). No thromboses were observed; coagulation was improved but not normalized. There were no treatment-emergent, clinically significant adverse events, and no abnormal hematology, chemistry, or pro-coagulant laboratory abnormalities throughout this study. Other preclinical studies in wild-type and hemophilia B dogs and in minipigs confirmed the lack of treatment‐emergent, clinically significant adverse events. Furthermore, there was no skin reaction at 24 h and 48 h in all minipigs following SQ dosing of dalcinonacog alfa [49, 50].

**A**: **WBCT**

**B**: **aPTT**

**C**: **FIX Antigen Levels**

**D**: **FIX Activity Levels**

**E**. **Correlation of FIX activity and antigen in plasma**

## Discussion

A subcutaneous FIX product with a longer half-life to prolong the protective hemostatic effect would potentially offer several benefits to patients with hemophilia B, including a reduced number of intravenous injections, consistent higher FIX activity levels, improved acceptance of prophylactic regimens and patient adherence, and enhanced quality of life [30]. It appears that the enhanced biological properties of dalcinonacog alfa may confer these benefits. The benefit of daily SQ dalcinonacog alfa is resultant high levels and is steady high levels likely sufficient to prevent spontaneous bleeds.

The intravenous pharmacokinetic profile of dalcinonacog alfa was similar to recombinant FIX but had 22-fold reduced mass when dosed using the same activity, as it has approximately 22-times greater potency [37]. Single-dose subcutaneous injections did not result in measurable antigen levels in the blood and we attribute this to the need to distribute factor to the extravascular compartment before blood levels can be detected. [51]. Daily subcutaneous dalcinonacog alfa, which has no alteration of the collagen-binding site located in the N-terminal Gla-domain, distributes freely in the extravascular compartment based on volume of distribution PK calculations, as does wild-type FIX. Four days of regular injections in dogs sufficiently demonstrated the effects of the bioavailability, potency, and prolonged effective half-life to reach activity levels sufficient to correct severe hemophilia to normal. Subcutaneous dalcinonacog alfa did not produce any clinical adverse events for the duration of the study. A limitation of canine models of inherited bleeding disorders is the development of antibodies to human proteins precluding long-term use. Preclinical animal studies cannot predict antibody development in humans [52]. Additional studies are required to monitor immunogenicity to dalcinonacog alfa. Although this study initially utilized a chromogenic activity assay, we subsequently learned that the activity of dalcinonacog alfa is best determined by a one-stage assay [44, 53] Results presented in Fig 3D thus represent those of the one-stage clotting assay. Interestingly, a significant correction in the WBCT and aPTT was observed within the first 24 hours after the first dose of 300 IU/kg dalcinonacog alfa, whereas the observable plasma levels of FIX activity were approximately 5% and negligible antigen was detected. These data suggest that dalcinonacog alfa is efficacious at correcting markers for normal coagulation at low plasma concentrations in keeping with more rapid and increased enzyme kinetics and activity.

Early individualized prophylactic therapy in individuals with hemophilia B has proven and profound medical benefits [11–16]. However, prophylaxis therapy needs to be effective, safe, convenient, and simple to utilize to improve acceptance by caregivers and individuals with hemophilia B [22–27].

## Conclusion

The data from this preclinical study demonstrate that regular subcutaneous administration of dalcinonacog alfa, resulting in high and prolonged circulating FIX activity, may provide a more effective and convenient treatment than current intravenous rFIX products. The increased potency of dalcinonacog alfa allows low-volume injection and attains meaningful antigen and activity levels in the blood. Moreover, no potential safety concerns were identified with dalcinonacog alfa. However, immunogenicity should be closely monitored in future studies with dalcinonacog alfa. Clinical trials have been initiated to evaluate the efficacy, PK, PD, bioavailability, and safety of daily subcutaneous treatment with dalcinonacog alfa for bleeding prophylaxis in adults with hemophilia B.

## Supporting information

S1 Table Single intravenous infusion of 50 IU/kg dalcinonacog alfa in hemophilia dogs and consequent blood levels, FIX activity and coagulation results. (DOCX)
