# Supplementary material for: Preclinical evaluation of a next-generation, subcutaneously administered, coagulation factor IX variant, dalcinonacog alfa
Source: PLoS One. 2020 Oct 28;15(10):e0240896. doi: 10.1371/journal.pone.0240896 (PMC7592742; doi:10.1371/journal.pone.0240896)
Supplement: S1 Table — (DOCX) [file pone.0240896.s001.docx]

## S1 Table. Single Intravenous Infusion of 50 IU/kg Dalcinonacog Alfa in Hemophilia Dogs and Consequent Blood Levels, FIX Activity and Coagulation Results

**A. Tony**

| **Day** | **Time** | **WBCT** | **PLT** | **WBC** | **HCT** | **Hb** | **aPTT (180 sec. incub. - Triniclot)** | **FIX activity** | **FIX antigen** | **F1+2** | **d-dimer** | **TAT** | **Fibrinogen** |
| --- | --- | --- | --- | --- | --- | --- | --- | --- | --- | --- | --- | --- | --- |
|  |  | **min** | **200-500 10^3/mm^3** | **6.0-17.0 10^3/mm^3** | **37-55%** | **12.0-18.0 g/dL** | **sec** | **%** | **%** | **ng/ml** | **ng/mL** | **µg/L** | **mg/dL** |
| 0 | Pre | >60.0 | 287 | 5.2 | 51.2 | 16.4 | 58.1 | <0.6 | <0.8 | 4.0 | 66 | 3.2 | 351 |
|  |  |  |  |  |  |  |  |  |  |  |  |  |  |
|  | 15 min | 24.5 | 294 | 5.3 | 48.4 | 15.6 | 28.8 | 8.4 | 2.4 | 4.3 | 70 | 3.2 | 350 |
|  | 1 hr |  |  |  |  |  | 28.8 | 10.6 | 2.0 | 4.2 | 77 | 3.3 | 332 |
|  | 4 hr | 23 |  |  |  |  | 29.8 | 10.2 | 2.2 | 4.8 | 50 | 3.3 | 347 |
|  | 8 hr | 27 | 298 | 7.2 | 49 | 16.1 | 30.1 | 9.2 | 2.0 | 2.5 | 271 | 4.9 | 324 |
|  | 12 hr |  |  |  |  |  | 31.4 | 6.0 | 1.6 | 0.9 | 162 | 3.5 | 345 |
| 1 | 24 hr | 24.5 | 239 | 6.4 | 46.6 | 14.8 | 33.4 | 4.6 | 1.2 | 2.9 | 119 | 4.0 | 367 |
| 2 | 48 hr | 26.5 | 262 | 7.5 | 47.2 | 15.3 | 33.4 | 4.1 | 0.9 | 5.4 | 43 | 3.3 | 366 |
| 3 | 72 hr | 26.5 | 264 | 6.9 | 47.3 | 15.1 | 33.4 | 1.9 | 0.9 | 5.5 | 43 | 3.4 | 364 |
| 4 | 96 hr | 29.5 |  |  |  |  | 34.2 | 1.2 | <0.8 | 4.1 | 69 | 3.5 | 338 |
| 5 |  |  |  |  |  |  | 38.9 | 0.833 | <0.8 | 4.1 | 99 | 3.3 | 338 |
| 6 |  |  |  |  |  |  | 39.9 | <0.6 | <0.8 | 2.0 | 0 | 3.6 | 334 |
| 7 |  | 44.5 | 305 | 6.6 | 50.4 | 15.7 | 38.8 | <0.6 | <0.8 | 3.4 | 24 | 3.5 | 310 |
| 8 |  | 41 |  |  |  |  | 42.2 | <0.6 | <0.8 | 4.2 | 108 | 3.8 | 372 |
| 9 |  | 46 |  |  |  |  | 44.5 | <0.6 | <0.8 | 2.7 | 32 | 3.3 | 338 |
| 10 |  | 52.5 |  |  |  |  | 43 | <0.6 | <0.8 | 17.6 | 105 | 3.8 | 373 |
| 11 |  | 48 |  |  |  |  | 46.4 | <0.6 | <0.8 | 8.8 | 52 | 5.6 | 407 |
| 12 |  |  |  |  |  |  | 44.9 | <0.6 | <0.8 | 7.3 | 97 | 4.1 | 390 |
| 14 |  | >60.0 | 265 | 7.5 | 50.8 | 16 | 46.1 | <0.6 | <0.8 | 9.1 | 59 | 3.6 | 410 |
| 15 |  | >60.0 |  |  |  |  | 47.4 | <0.6 | <0.8 | 9.9 | 80 | 4 | 483 |
| 16 |  | >60.0 |  |  |  |  | 52 | <0.6 | <0.8 | 12.4 | 53 | 3.6 | 472 |
| 17 |  | >60.0 |  |  |  |  | 53.4 | <0.6 | <0.8 | 12.3 | 37 | 3.3 | 431 |
| 18 |  | >60.0 |  |  |  |  | 52 | <0.6 | <0.8 | 5.1 | 159 | 3.2 | 440 |

**B. Bennett**

| **Day** | **Time** | **WBCT** | **PLT** | **WBC** | **HCT** | **Hb** | **aPTT (180 sec. incub. - Triniclot)** | **FIX activity** | **FIX antigen** | **prothrombin F1+2** | **d-dimer** | **TAT** | **fibrinogen** |
| --- | --- | --- | --- | --- | --- | --- | --- | --- | --- | --- | --- | --- | --- |
|  |  | **min** | **200-500 10^3/mm^3** | **6.0-17.0 10^3/mm^3** | **37-55%** | **12.0-18.0 g/dL** | **sec** | **%** | **%** | **ng/ml** | **ng/ml** | **μg/dl** | **mg/dl** |
| 0 | Pre | >60.0 | 304 | 7.2 | 55.2 | 17.7 | 55.8 | <0.6 | <0.8 | 2.9 | 131 | 3.6 | 324 |
|  | 15 min | 20.5 | 303 | 7.1 | 54.4 | 17.1 | 28.9 | 6.0 | 1.7 | 3.9 | 192 | 10.3 | 308 |
|  | 1 hr |  |  |  |  |  | 28.5 | 7.2 | 1.7 | 4.5 | 96 | 4.1 | 308 |
|  | 4 hr | 21.5 |  |  |  |  | 28.8 | 4.5 | 1.4 | 1.9 | 124 | 6.2 | 295 |
|  | 8 hr | 21 | 344 | 7.8 | 54.7 | 17.5 | 30.4 | 4.1 | 1.2 | 0.5 | 155 | 3.5 | 291 |
|  | 12 hr |  |  |  |  |  | 29.6 | 3.8 | 1.1 | <0.1 | 99 | >99.9 | 297 |
| 1 | 24 hr | 22 | 296 | 6.7 | 54.7 | 17.1 | 29.8 | 3.5 | 0.8 | 3.6 | 170 | 3.9 | 331 |
| 2 | 48 hr | 21 | 403 | 9.7 | 56.6 | 18 | 34.3 | 1.2 | <0.8 | <0.1 | 213 | 3.6 | 279 |
| 3 | 72 hr | 23.5 | 341 | 8.9 | 56.1 | 18.1 | 36.4 | 0.8 | <0.8 | 2.0 | 92 | 4.0 | 307 |
| 4 | 96 hr | 23 |  |  |  |  | 36.2 | <0.6 | <0.8 | 0.2 | 151 | 3.7 | 280 |
| 5 |  |  |  |  |  |  | 37.8 | <0.6 | <0.8 | 0.3 | 138 | 4.3 | 277 |
| 6 |  |  |  |  |  |  | 37.5 | <0.6 | <0.8 | 1.9 | 97 | 3.7 | 253 |
| 7 |  | 28 | 296 | 7.1 | 55.7 | 17.9 | 40.5 | <0.6 | <0.8 | 1.9 | 72 | 5.0 | 242 |
| 8 |  | 25 |  |  |  |  | 39.6 | <0.6 | <0.8 | 2.2 | 133 | 3.7 | 295 |
| 9 |  | 26.5 |  |  |  |  | 44.1 | <0.6 | <0.8 | 4.2 | 310 | 3.5 | 341 |
| 10 |  | 29 |  |  |  |  | 41.2 | <0.6 | <0.8 | 9.2 | 142 | 4.0 | 415 |
| 11 |  | 35 |  |  |  |  | 48.5 | <0.6 | <0.8 | 13.8 | 159 | 3.3 | 658 |
| 12 |  |  |  |  |  |  | 47.7 | <0.6 | <0.8 | 4.1 | 152 | 3.6 | 536 |
| 14 |  | 37 | 323 | 8.1 | 54.5 | 17.7 | 46.6 | <0.6 | <0.8 | 7.7 | 98 | 3.6 | 386 |
| 15 |  | 41 |  |  |  |  | 45.4 | <0.6 | <0.8 | 9.3 | 110 | 3.7 | 378 |
| 16 |  | 38.5 |  |  |  |  | 49.8 | <0.6 | <0.8 | 7.3 | 92 | 8.2 | 353 |
| 17 |  | >60.0 |  |  |  |  | 49 | <0.6 | <0.8 | 6.4 | 122 | 3.9 | 301 |
| 18 |  | >60.0 |  |  |  |  | 48.9 | <0.6 | <0.8 | 1.4 | 120 | 3.9 | 299 |

aPTT, activated partial thromboplastin time; FIX, factor IX; Hb, hemoglobin; incub, incubation; kg, Min, minutes; sec, second; WBCT, whole blood clotting time.
